# O02 Nitrofurantoin: what is the evidence for current guidance?

**DOI:** 10.1093/jacamr/dlad077.002

**Published:** 2023-08-02

**Authors:** Eleanor Kashouris, Amelia Joseph, Tom Lewis

**Affiliations:** Department of Sociology and Criminology, Freeman Building, University of Sussex, Falmer, BN1 9RH, UK; Department of Clinical Microbiology, Nottingham University Hospitals NHS Trust, Nottingham, NG7 2UH, UK; Department of Microbiology, Royal Devon University Hospital, Barnstaple, EX31 4JB, UK

## Abstract

In this abstract, we review current approaches to the management of uncomplicated urinary tract infection (UTI) and begin to explore alternative approaches to antibiotic prescribing. We review clinical guidelines on the use of nitrofurantoin internationally to capture the level of variation in evidence-based guidance. We find that the evidence base has been interpreted in very different ways. UK guidelines from NICE and the Scottish Intercollegiate Guidelines Network (SIGN) are unusual in promoting short (3 day) courses of nitrofurantoin. We find little direct evidence to support this, and that this guidance has been extrapolated from studies based on other agents. ‘Short’ courses of antibiotics aim to provide optimum balance between providing effective treatment whilst reducing selective pressure driving resistance amongst colonizing microbial flora. Three days of nitrofurantoin may indeed be a useful intervention in a large group of patients. Longer courses of nitrofurantoin still demonstrate considerable rates of treatment failure. The concept of a course length in itself is limited. The variation between recommendations internationally reflects both the uncertain foundations upon which evidence-based guidelines are written, and the limits of what can be safely concluded from evidence. We join others in pointing out there may be ways of customizing duration of therapy to the patient’s response in primary care settings. There is a need for a more patient-focused approach to uncomplicated UTI, including better safety-netting and (currently lacking) guidance on best practice in the common clinical scenario of treatment failure.

